# 

**Published:** 1934

**Authors:** 


### The Medical Library of the University of Bristol,

WITH WHICH IS INCORPORATED

**The Library of the Bristol Medico-Chirurgical Society.**

---

*The following donations have been received since the publication of the list in September,* 1934.

December, 1934.

| | | |
|---|---|---|
| J. Michell Clarke Fund (1) | .. .. .. | 29 volumes. |
| Professor E. W. Hey Groves (2) | .. .. | 5 ,, |
| Dr. Iwao Matsuo (3) | .. .. .. .. | 1 volume. |
| Dr. C. Bruce Perry (4) | .. .. .. .. | 1 ,, |
| Royal College of Surgeons of England (5) | .. | 1 ,, |

Unbound periodicals have also been received from Professor E. W. Hey Groves, Dr. C. Bruce Perry and Dr. J. Odery Symes.

### THE ONE HUNDRED AND FORTY-NINTH LIST OF BOOKS.

The figures in round brackets refer to the figures after the names of the donors. The books to which no such figures are attached have either been bought from the Library Fund or received through the *Journal.*

| | | | |
|---|---|---|---|
| **Abt, I. A.** .. .. | *Pediatrics* .. .. .. .. .. .. .. .. .. .. .. | | [1932] |
| **Azémar, A.** .. .. | *Le paragangliome surrénal* .. .. .. | (4) | 1930 |
| **Bastos, M.** .. .. | *La Osteosintesis* .. .. .. .. .. .. .. .. .. | (2) | 1932 |
| **Bouman, L.** .. .. | *Diffuse Sclerosis* .. .. .. .. .. .. .. .. .. .. | | 1934 |
| **Boyd, W.** .. .. | *Textbook of Pathology* .. .. .. | 2nd Ed. | 1934 |
| **Brailsford, J. F.** .. | *Radiology of Bones and Joints* .. .. | (1) | 1934 |
| **Campbell, D.** .. .. | *Handbook of Therapeutics* .. .. | 2nd Ed. | 1934 |
| **Cantarow, A.** .. .. | *Calcium Metabolism* .. .. .. .. | 2nd Ed. (1) | 1933 |
| **Catterina, A.** .. .. | *L'opération de Bassini pour la hernie inguinale* | | 1934 |
| **Chopra, R. N.** .. | *Indigenous Drugs of India* .. .. .. .. .. | | 1933 |
| **Clark, A. J.** .. .. | *Applied Pharmacology* .. .. .. | 5th Ed. | 1933 |
| **Clark, W. E. Le G.** | *Early Forerunners of Man* .. .. .. | (1) | 1934 |
| **Colwell, H. A., and Russ, S.** | *X-ray and Radium Injuries* .. | (1) | 1934 |
| **Dore, S. E., and Franklin, J. L.** | *Diseases of the Skin* .. .. .. | | 1934 |
| **Findlay, G. M.** .. | *Recent Advances in Chemotherapy* .. .. .. | | 1930 |
| **Fleming, A., and Petrie, G. F.** | *Recent Advances in Vaccine Serum Therapy* .. .. .. .. .. .. .. .. .. | (1) | 1934 |
| **Fraser, J. E.** .. .. | *Anatomy of the Skeleton* .. .. | 3rd Ed. (1) | 1933 |
| **Furniss, A.** .. .. | *Ultra-Violet Therapy* .. .. .. .. .. | (1) | 1931 |
| **Gadelius, B.** .. .. | *Human Mentality in the Light of Psychiatric Experience* .. .. .. .. .. .. .. .. | (1) | 1933 |

**Gilford, H.** .. .. *The Cancer Problem* .. .. .. .. .. .. .. 1934
**Gould, G. M.** .. .. *Pocket Pronouncing Medical Dictionary* 10th Ed. 1934
**Graham, S., and Morris, N.** *Acidosis and Alkalosis* .. .. .. (1) 1933
**Gray, H.** .. .. .. *Anatomy, Descriptive and Applied* 25th Ed. (1) 1932
**Greenwood, M.** .. *Epidemiology, Historical and Experimental* (1) 1932
**Griffith, J. P. C., and Mitchell, A. G.** *Diseases of Infants* .. .. (1) 1933
**Grinker, R. R.** .. *Neurology* .. .. .. .. .. .. .. .. .. (1) [1934]
**Hammond, T. E.** .. *The Constitution and its Reaction in Health* 1934
**Hornibrook, E. A.** *Stand Up and Slim Down* .. .. 6th Ed. 1934
**Inkster, L.** .. .. *The Treatment of Functional Nerve Cases by the Method of Neuro-Induction* .. .. .. 1933
**Jameson, W. W., and Parkinson, G. S.** *A Synopsis of Hygiene* 4th Ed. (1) 1934
**Johnston, T. B.** .. *Synopsis of Regional Anatomy* 3rd Ed. (1) 1934
**Keith, A.** .. .. *Human Embryology and Morphology* 5th Ed. (1) 1933
**Kirschner, M.** .. .. *Operative Surgery. The Abdomen and Rectum* Trans. by Ravdin .. .. .. .. (1) [1933]
**Legge, T.** .. .. *Industrial Maladies* .. .. .. .. .. .. .. .. 1934
**Leschke, E.** .. .. *Clinical Toxicology.* Trans. by C. P. Stewart and O. Dorrer .. .. .. .. .. (1) 1934
**Magnus, G.** .. .. *Frakturen u. Luxationen* .. .. 2nd Ed. (2) 1933
**Marlin, T.** .. .. *Manipulative Treatment* .. .. .. .. .. .. 1934
**Matsuo, I.** .. .. *Biologische Untersuchungen über Farbstoffe* 1 Bd. (3) 1934
**Mennell, J. B.** .. *Physical Treatment by Movement, Manipulation and Massage* .. .. .. .. 3rd Ed. (1) 1934
**Miller, A.** .. .. *Modern Advances in Diseases of the Throat* .. 1934
**Netto, N. R.** .. .. *Acçao do tanaceto sobre a mortricidade do tracto digestivo* .. .. .. .. .. .. (2) [1933]
**New York Academy of Medicine.** *Maternal Mortality in New York* (1) 1933
**Nordlund, U.** .. .. *Über die sog. primäre . . . streptokokken-peritonitis* .. .. .. .. .. .. .. .. 1933
**Norris, G. W., and Landis, H. R. M.** *Diseases of the Chest* 5th Ed. (1) 1933
**Paul, N.** .. .. .. *Cutaneous Neoplasms* .. .. .. .. .. (1) 1933
**Payr, E.** .. .. .. *Gelenksteifen u. Gelenkplastik I. Teil.* .. (2) 1934
**Peters, J. P., and Van Slyke, D. D.** *Quantitative Clinical Chemistry* 2 vols. 1931–1932
**Reynolds, F. N.** .. *The Relief of Pain in Childbirth* .. .. (1) [1934]
**Ribeiro, E. B.** .. *Aspectos cirurgicos da caseose dos nervos na lepra* .. .. .. .. .. .. .. .. .. .. (2) 1934
**Robson, J. M.** .. *Recent Advances in Sex and Reproductive Physiology* .. .. .. .. .. .. .. .. 1934
**Rocaz, C.** .. .. *Pink Disease.* Trans. I. J. Wood .. .. (1) 1933
**Rogers, J. S.** .. .. *Physics for Medical Students* .. .. .. .. 1933
**Rose, W., and Carless, A.** *Manual of Surgery* [ed.] by C. P. G. Wakeley and J. B. Hunter. 2 vols. 14th. Ed. (1) 1933
**Roxburgh, A. C.** .. *Common Skin Diseases* .. .. .. 2nd Ed. 1934
**Sollmann, T.** .. .. *Manual of Pharmacology* .. .. 4th Ed. (1) 1932

| | | | |
|---|---|---|---|
| **Sorsby, A.** .. .. | *A Short History of Ophthalmology* .. .. .. | | 1933 |
| **Stewart, T. J.** .. | *Notes on Milk* .. .. .. .. .. .. .. .. .. | | 1934 |
| **Ten Teachers** .. .. | *Diseases of Women* .. .. .. .. .. | 5th Ed. | 1934 |
| **Thomas, E. W. C.** | *A Synopsis of Hygiene* .. .. .. .. .. .. | | 1934 |
| **Thompson, C. J. S.** | *Lord Lister* .. .. .. .. .. .. .. .. .. | (1) | 1934 |
| **Tidy, H. L.** .. .. | *A Synopsis of Medicine* .. .. .. .. | 6th Ed. | 1934 |
| **Tidy, N. M.** .. .. | *Massage and Remedial Exercises* .. | 2nd Ed. | 1934 |
| **Vaughan, J. M.** .. | *The Anœmias* .. .. .. .. .. .. .. .. .. | | 1934 |
| **Wakeley, C. P. G.** | *Aids to Operative Surgery* .. .. .. .. | 2nd Ed. | 1934 |
| *Wellcome Research Institution and the Affiliated Laboratories* .. .. | | | 1934 |
| **Williams, L.** .. .. | *Aids to Obstetrics* .. .. .. .. .. | 10th Ed. | 1934 |
| **Willis, R. A.** .. .. | *The Spread of Tumours in the Human Body* | (1) | 1934 |

TRANSACTIONS, REPORTS, JOURNALS, ETC.

| | | |
|---|---|---|
| Annual Report of the Chief Inspector of Factories, etc. .. | | 1924–onwards |
| Medical Directory, 1935 .. .. .. .. .. .. .. .. .. .. .. .. .. | | 1935 |
| Nature of Disease Journal. Vol. III. .. .. .. .. .. .. .. .. .. | | 1934 |
| Quarterly Cumulative Index Medicus. Vol. 15 .. .. .. .. .. .. | | 1934 |
| Royal College of Surgeons of England. Calendar, 1934 .. .. | (5) | 1934 |
| Surgical Clinics of North America. Vol. XIV., Nos. 4 and 5 .. | | 1934 |
| Westminster Hospital Reports. Vol. XXII. (1929–1933) .. .. | | 1934 |

### Publications Received

From Edward Arnold & Co :

*Anæsthesia and Analgesia in Labour.* By K. L. Lloyd-Williams, M.D., B.S. Price 5s.

*The Treatment of Common Female Ailments.* By F. J. McCann, M.D. 3rd Ed. Price 12s. 6d.

*Diseases of Children.* By A. E. Garrod, D.M., F. E. Batten, M.D., M.A., and H. A. Thursfield, D.M., M.A. 3rd Ed. Edited by H Thursfield and D. Paterson. Price £2 10s. 0d.

From John Bale, Sons & Danielsson, Ltd. :

*Melancholia in Everyday Practice.* By E. L. Hopewell-Ash, M.D. Price 7s. 6d.

From Cassell & Co. Ltd. :

*Surgical Applied Anatomy.* By F. Treves, Bart. 9th Ed. Revised by C. C. Choyce. Price 14s.

From William Heinemann, Ltd. :

*The Rheumatic Diseases.* By G. D. Kersley, M.A., M.D. Price 6s.

*High Blood Pressure.* By J. F. H. Dally, M.A., M.D. 3rd Ed. Price 15s.

*Radium and Cancer.* By H. S. Souttar, D.M., M.Ch. Price 21s.

*What of the Child ?* By A. Kefalas, M.A., M.B., Ch.B. Price 5s.

From H. K. LEWIS & CO. LTD. :

*Principles in the Treatment of Inflammation.* By T. E. HAMMOND. Price 10s. 6d.

*Materia Medica for Nurses.* By A. M. CRAWFORD, M.D. 3rd Ed. Price 3s. 6d.

*The Etiology and Treatment of Spasmodic Bronchial Asthma.* By H. G. OLIVER, M.D. Price 3s. 6d.

*Westminster Hospital Reports.* Vol. XXII. 1929–1933. Price 7s. 6d.

From WILHELM MAUDRICH :

*Periodic Fertility and Sterility in Woman.* By H. KNAUS. Price RM. 15.

From OXFORD UNIVERSITY PRESS :

*B.C.G. Vaccine.* By K. N. IRVINE, D.M., M.A., B.Ch. Price 5s.

*Head Injuries.* By L. B. RAWLING, M.B., B.Ch. Price 7s. 6d.

From THE STUDENT CHRISTIAN MOVEMENT PRESS :

*Right Marriage.* By F. R. BARRY, C. MULLINS, and D. WHITE, M.A., M.D. Price 6d.

From JOHN WRIGHT & SONS LTD. :

*British Journal of Surgery.* Vol. XXII., No. 86. October, 1934. Price 12s. 6d.

*An Atlas of the Common Skin Diseases.* By H. C. G. SEMON, M.A., M.D. Price £2 2s. 0d.

*Synopsis of Surgical Anatomy.* By A. L. McGREGOR, 2nd Ed. Price 17s. 6d.

*The British Journal of Surgery.* Vol. XXII., No. 85. July, 1934. Price 12s. 6d.

From WELLCOME FOUNDATION, LTD. :

*The Wellcome Research Institution, London. Exhibits at the Chicago Exposition, 1934.*

### Local Medical Notes

**University of Bristol.**—Chair of Anatomy : The vacant Chair of Anatomy has been filled by the appointment of Dr. Samuel Ernest Whitnall, F.R.S. (Canada), D.M., formerly Robert Reford Professor of Anatomy in McGill University.

Chair of Medicine : The vacant Chair of Medicine has been filled by the appointment of C. Bruce Perry, M.D., M.R.C.P.

Chair of Obstetrics : The vacant Chair of Obstetrics has been filled by the appointment of H. J. Drew Smythe, M.D., M.S., F.R.C.S.

Professor Geoffrey Hadfield has resigned the Chair of Pathology, on accepting the St. Bartholomew's Hospital Chair of Pathology in the University of London.

EXAMINATION RESULTS.

Students of the University have recently passed the following examinations :—

M.D.—J. C. Batt.

M.B., Ch.B.—*Supplementary First Examination. Pass :* B. C. E. Aldous, Ruth Appleby, Dorothy M. Ayre, Mary Crago, Sara M. Field-Richards, E. M. Grace, C. J. R. Jacob, Rosemary W. Knowles.

M.B., Ch.B.—*Final Examination.* (*Part I.*) *Pass :* Monica L. Hawkins, B. Isserlin (with distinction in Pathology), J. S. W. Little, N. R Matheson (with distinction in Materia Medica, Pharmacy, Pharmacology, Therapeutics, and Forensic Medicine and Toxicology), E. Philipp, L. A. Schnipelsky, E. Want, E. L. Ward.

M.B., Ch.B.—*Final Examination.* (*Part II.*) *Pass :* Marion L. Tryon (*with Second Class Honours*), R. A. Mathews (*with Second Class Honours*), R. D. Bodman, J. N. Heales, F. J. W. Lewis, A. J. Matheson, Mary G. Thomas, H. James.

B.D.S.—*Second Examination. Pass :* F. P. Ewens.

B.D.S.—*Third Examination. Pass : In Dental Mechanics and Dental Metallurgy :* A. N. Brimelow, C. C. B. Plumley, P. R. Quartley. *In Dental Materia Medica :* C. A. Blanden.

L.D.S.—*Supplementary Preliminary Science. Pass :* C. F. Howell, Peggy M. Rooke, G. H. John, R. V. Wood.

L.D.S.—*First Professional Examination. Pass :* L. K. A. S. Oxley, J. B. Inverdale, J. C. F. Rolls, J. G. Windmill.

L.D.S.—*Second Professional Examination. Pass : In Dental Mechanics and Dental Metallurgy :* J. G. Coates, C. L. Read, A. J. Staple. *In Dental Materia Medica :* D. E. Royal. *In Dental Mechanics and Dental Metallurgy :* M. J. Banbury, R. A. Hilton, T. John.

**Conjoint Board.**—M.R.C.S., L.R.C.P.—*Pass :* R. T. Moore (*Elementary Biology*), Jane Mackintosh (*Physiology*), Margaret E. M. Slater (*Physiology*), E. J. Lace (*Pharmacology and Materia Medica*), T. H. Taylor (*Pathology*).

L.D.S., R.C.S.—*Final Examination.* (*Part II.*) *Pass :* J. H. G. Morris, J. W. E. Snawdon.

### Index

Abscess of the brain.—E. Watson-Williams, 231.
Adams, A. W.—Diagnosis of swellings of the long bones, with a case of periosteal sarcoma of the femur, 117.
Appointments—
Critchley, M.—Hunterian Professor, Royal College of Surgeons, 204.
Hadfield, G.—Professor of Pathology, University of London, 289.
Perry, C. Bruce.—Professor of Medicine, University of Bristol, 289.
Smythe, H. J. Drew.—Professor of Obstetrics, University of Bristol, 289.
Whitnall, S. E. — Professor of Anatomy, University of Bristol, 289.
Atrophic Rhinitis and Goitre, Association between.—E. Watson-Williams, 89.

Bicentenary of Royal Imfirmary, 280.

Bile, Expulsion of.—R. J. Brocklehurst, 131.

Bodman, F.—Nervous disorders in general practice, 47.

Brain, Abscess of.—E. Watson-Williams, 231.

Brocklehurst, R. J.—Expulsion of bile, 131.

Bush, G. B.—The clinical importance of the intervertebral discs, with special reference to nuclear prolapses, 173.

Cave, E. J.—Obituary, 73.

Cerebral dermoid.—P. Phillips and D. M. Stone, 247.

Chambers, E. R.—Congenital word-blindness, 41.

Childhood, Heart disease in.—F. J. Poynton (Long Fox Memorial Lecture), 205.

Coates, V. M.—Obituary, 281.

Colston Medical Research Fellowships, 153.

Congenital word - blindness.—E. R. Chambers, 41.

Cridland, A. B.—Obituary, 197.

Curriculum, Reform of.—By " A Medical Student," 253.

Dermoid, Cerebral.—P. Phillips and D. M. Stone, 247.
Dunhill, Sir T. P.—The place of surgery in hyperthyroidism, 155.

Examination results, 79, 153, 204, 290.
Expulsion of bile.—R. J. Brocklehurst, 131.

Fawcett, Professor E.—Dinner to, 193.
Femur, Periosteal sarcoma of.—A. W. Adams, 117.
Focal infection.—A. J. M. Wright, 109.

Goitre and atrophic rhinitis, Association between.—E. Watson-Williams, 89.

Hadfield, G.—Appointment to Chair of Pathology at St. Bartholomew's Hospital, 289.
Harris, H. E.—Looking back, 1.
Heart disease in childhood.—F. J. Poynton (Long Fox Memorial Lecture), 205.
Hyperthyroidism, The place of surgery in.—Sir T. P. Dunhill, 155.

Infirmary, Royal, Bicentenary, 280.
Index to Volumes 1 to 50, 70.
Intervertebral discs, Clinical importance of.—G. B. Bush, 173.

Jones, T.—Obituary, 148.

King, W. W.—Obituary, 200.

Library Notes, 75, 150, 201, 286.
Liverpool Medical School Centenary, 147.
Local Medical Notes, 79, 153, 204, 289.
Long bones, Diagnosis of swellings of.—A. W. Adams, 117.
Long Fox Memorial Lecture.—F. J. Poynton.—Some aspects of heart disease in childhood, 205.

Medical curriculum, Reform of.—By " A Medical Student," 253.
" Medical Student."—Reform of the medical curriculum, 253.
Mules, Mary S.—Obituary, 282.

Neild, N.—Obituary, 198.
Nervous disorders in general practice.—F. Bodman, 47.
Nuclear prolapses (Intervertebral discs).—G. B. Bush, 173.

Obituaries—
E. J. Cave, 73.
V. M. Coates, 281.
A. B. Cridland, 197.
T. Jones, 148.
W. W. King, 200.
Mary S. Mules, 282.
N. Neild, 198.
J. L. Pearce, 147.
C. Randolph, 71.
Orr-Ewing, H. J.—Medical practice in Palestine, 163.

Palestine, Medical practice in.—H. J. Orr-Ewing, 163.
Parker, G.—Hon. degree, LL.D., 194.
Pearce, J. L.—Obituary, 147.
Periosteal sarcoma of femur.—A. W. Adams, 117.
Perry, C. B.—Appointment to Chair of Medicine at University of Bristol, 289.
Phillips, P., and Stone, D. M.—Cerebral dermoid, 247.
Poynton, F. J.—Some aspects of heart disease in childhood (Long Fox Memorial Lecture), 205.
Presidential Address, H. E. Harris.—Looking back, 1.
Prolapses, Nuclear (Intervertebral discs).—G. B. Bush, 173.

Randolph, C.—Obituary, 71.
Reform of the medical curriculum.—By "A Medical Student," 253.
Resignations—
Professor R. S. S. Statham, 196.
Professor Geoffrey Hadfield, 289.
Reviews of Books, 59, 139, 183, 265.
Rhinitis, atrophic, and goitre, Association between.—E. Watson-Williams, 89.
Royal Infirmary, Bicentenary, 280.
Royal Sanitary Institute, Health Congress at Bristol, 195.

Sarcoma, periosteal, of the femur.—A. W. Adams, 117.
Smythe, H. J. D.—Appointment to Chair of Obstetrics at University of Bristol, 289.
Societies—
Bristol Medico - Chirurgical Society, 73, 149, 283.
Statham, R. S. S.—Vaginal discharge, 27.
Resignation of Chair of Obstetrics, 196.
Stone, D. M.—See Phillips, P. and Stone, D. M.
Surgery in hyperthyroidism, The place of.—Sir T. P. Dunhill, 155.

Townsend, Miss F. M.—Resignation as Chairman of Winford Orthopædic Hospital, 70.

Vaginal discharge.—R. S. S. Statham, 27.

Watson-Williams, E.—
Association between goitre and atrophic rhinitis, 89.
Abscess of the brain, 231.
Watson - Williams, P. — Hon. Degree, M.D., 194.
Whitnall, S. E.—Appointment to Chair of Anatomy at University of Bristol, 289.
Winford Orthopædic Hospital, Resignation of Chairman.—Miss F. M. Townsend, 70.
New Wards, 279.
Word - blindness, Congenital.—E. R. Chambers, 41.
Wright, A. J. M.—Focal infection, 109.

